# How New and Expecting Fathers Engage With an App-Based Online Forum: Qualitative Analysis

**DOI:** 10.2196/mhealth.9999

**Published:** 2018-06-18

**Authors:** Becky K White, Roslyn C Giglia, Jane A Scott, Sharyn K Burns

**Affiliations:** ^1^ School of Public Health Curtin University Perth Australia; ^2^ Telethon Kids Institute University of Western Australia Perth Australia; ^3^ Collaboration for Evidence, Research and Impact in Public Health Curtin University Perth Australia

**Keywords:** mHealth, mobile app, breastfeeding, fathers, online communities

## Abstract

**Background:**

Breastfeeding is important for infants, and fathers are influential in supporting their partner in their decision to breastfeed and how long they breastfeed for. Fathers can feel excluded from traditional antenatal education and support opportunities but highly value social support from peers. Online health forums can be a useful source of social support, yet little is known about how fathers would use a conversation forum embedded in a breastfeeding-focused app. Milk Man is a mobile app that aimed to increase paternal support for breastfeeding using a range of strategies, including a conversation forum.

**Objective:**

The aim of this study was to examine how fathers used a breastfeeding-focused conversation forum contained within a mobile app throughout the perinatal period.

**Methods:**

A qualitative analysis of comments posted by users in the online forum contained within the Milk Man app was conducted. The app contained a library of information for fathers, as well as a conversation forum. Thematic analysis was used to organize and understand the data. The NVivo 11 software package was used to code comments into common nodes, which were then organized into key themes.

**Results:**

In all, 208 contributors (35.5% [208/586] of those who had access to the app) posted at least once within the forum. In total, 1497 comments were included for analysis. These comments were coded to 3799 individual nodes and then summarized to 54 tree nodes from which four themes emerged to describe how fathers used the app. Themes included seek and offer support, social connection, informational support provision, and sharing experiences. Posting in the forum was concentrated in the antenatal period and up to approximately 6 weeks postpartum.

**Conclusions:**

These data show that fathers are prepared to use a breastfeeding-focused online forum in a variety of ways to facilitate social support. Fathers can be difficult to reach in the perinatal period, yet engaging them and increasing social support is important. This research demonstrates the acceptability of an innovative way of engaging new and expecting fathers.

## Introduction

### Breastfeeding

Breastfeeding is of key importance to public health. The World Health Organization recommends that babies are exclusively breastfed to 6 months and for breastfeeding, supplemented with appropriate complementary foods, to continue for 2 years and beyond [1]. There are numerous, well-evidenced health benefits for infants and mothers, including a reduction in risk of a number of infections, sudden infant death syndrome and obesity in later life for infants [2,3], and protection against ovarian and breast cancer and improved bone remineralization in mothers [3,4].

Despite the recommendations and the benefits of breastfeeding, only about 15% of Australian infants are exclusively breastfed to 5 months of age [5]. There are many factors that impact breastfeeding [6,7], including the support of fathers. Targeting breastfeeding interventions toward fathers can positively impact breastfeeding duration [8]. Although research shows most fathers are supportive of their partners breastfeeding [7,9], there are a number of factors that impact the support they can offer [10-12]. These include the following:

1. Social support: fathers not receiving enough social support with pregnancy and early parenting
2. Knowledge: fathers want more knowledge about breastfeeding, pregnancy, and early parenting
3. Empowerment: a lack of understanding and recognition of the paternal role in breastfeeding
4. Barriers: specific barriers such as public breastfeeding and bonding postponement.

### Social Support Via Online Health Forums

Increased levels of social support can have benefits for participants in terms of their mental and physical health [13]. The facilitation of social support via online health communities (OHCs) is an area of increasing research interest [14]. Seeking social support can be a key reason that people participate in OHCs, and there are benefits for those who receive social support online [15]. One of the benefits of OHCs is that participants can use them in different ways and that access to the information is available whenever the user wants. Some participants will use an OHC to actively connect with others, whereas others will prefer to simply observe and receive the information [16].

Participation in OHCs can offer both benefits and drawbacks to users. The availability of access whenever the user requires, as well as the ability for online forums to facilitate bringing people together who may share an interest or health issue but are geographically distant, can be a significant benefit [17]. Social networks can also offer a level of anonymity, which may make it easier for people to seek support, especially in circumstances where they may not feel comfortable talking to people they know [18]. People seeking to lose weight, for example, could join a support group of people from around the country or even worldwide that share their specific goal. Same sex attracted young adults in rural communities could find peers online. Parents struggling with their children’s behavior could find others in the same situation.

Although there are positive aspects of connecting people, technology also comes with risks. The anonymity which can enable sharing can also provide opportunity and impunity for people to attack and bully others [19]. In terms of health information seeking, some studies report it can also lead to misinformation being sourced and shared [20]. However, other studies have found community-moderated OHCs can maintain a high quality of health information [21].

In their analysis of a large, popular breast cancer OHC, Wang et al found participants used the forum in a number of different ways [14]. Informational support, including seeking and providing information, was the most popular way support was facilitated. Companionship, which is the discussion of other issues rather than the actual health issue, was the key factor in retaining engagement in the online community over time [14].

### Reaching New Fathers Via Online Forums

In Australia, the penetration of mobile devices has been increasing exponentially and offers opportunity to reach people with health interventions. In 2016, Deloitte estimated that approximately 84% of Australian adults owned a smartphone, which represents a nearing of peak market saturation [22]. A recent survey of Australian smartphone users (n=14,000) found that 40% of respondents used their mobile phone for a minimum of 3 hours each day, and an additional 47% used it for between 1 and 2 hours a day [23].

Some of the benefits offered by online forums are particularly pertinent when developing social support opportunities for fathers. Social support has been shown to have a buffering effect on parental stress [24]. In preparing for the birth of their child, fathers can feel isolated and feel that antenatal education is not inclusive of them [10]. In the perinatal period, fathers highly value social support, and support from peers is particularly sought after [12].

Participants in the Fathers Infant Feeding Initiative study, a fathers-focused randomized controlled trial that aimed to increase paternal support for breastfeeding, identified barriers to their access to support services [10,11]. These included accessibility and flexibility (particularly the need to balance work commitments), and the use of information technology was one recommendation to overcome these barriers. As fathers have reported feeling disempowered about their role in breastfeeding [25,26], the relative anonymity associated with online forums may further facilitate fathers actively participating in conversations about breastfeeding.

### Milk Man App

**Figure 1:**
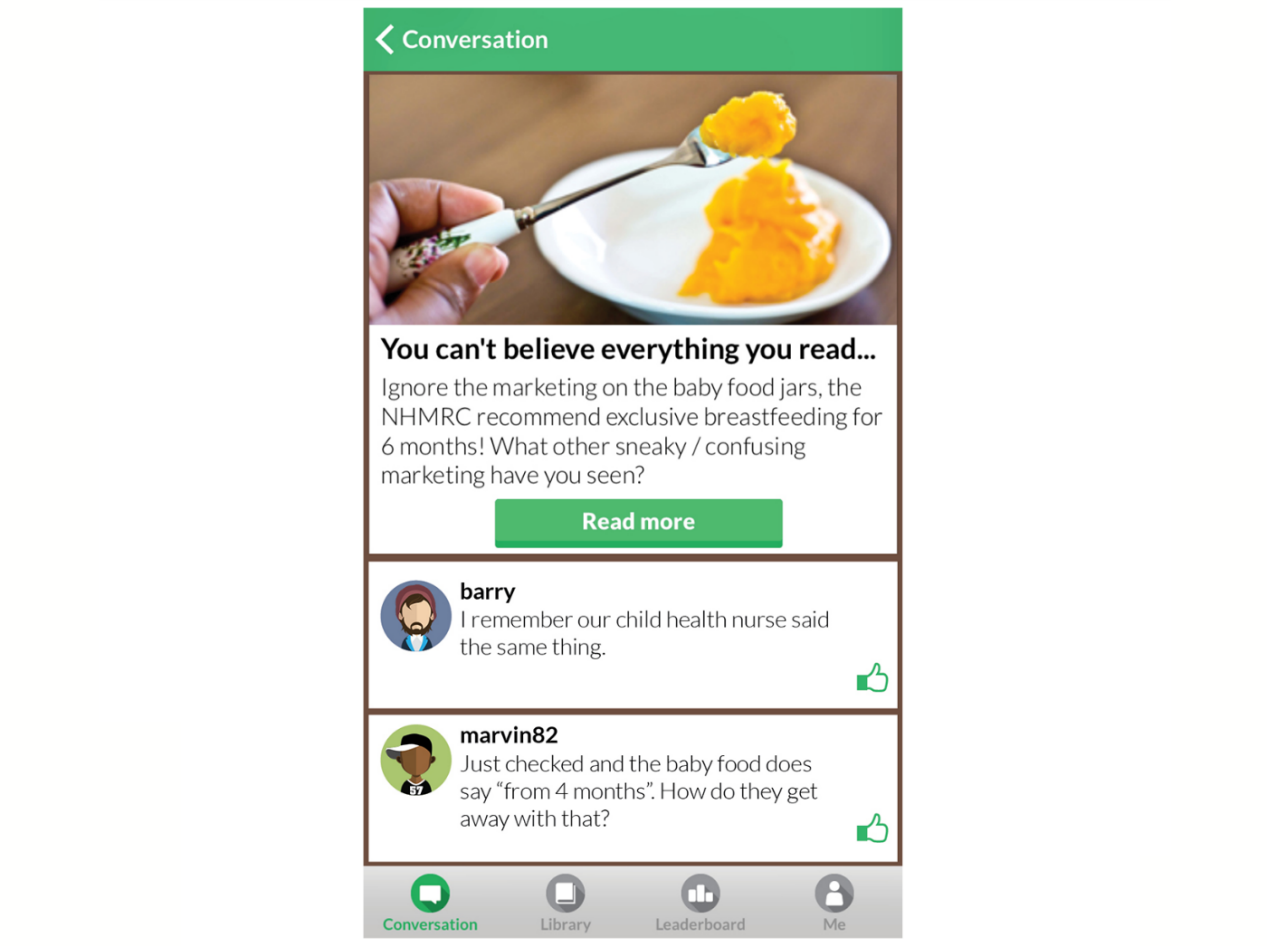
Example of a topic posed within the conversation forum.

The Milk Man app was designed to engage fathers in information and conversation about breastfeeding, with an aim to increase the support they offered to their breastfeeding partners. The development and trialing of the Milk Man app has been previously described [27,28]. A key component was the *conversation*, a facilitated forum whereby participants were posed questions via a series of topics and provided opportunities to comment. Figure 1 shows an example of a conversation topic within the forum. When fathers first signed up to use the app in the antenatal period, they were grouped depending on when their baby was due.

This enabled time-relevant questions to be posed and for the opportunity to talk to other fathers at a similar stage of pregnancy or early parenthood. New content was added to the app from when fathers signed up until their babies were approximately 26 weeks of age. The topics were designed to be either timely in relation to infant milestones in the perinatal stage or to focus on community building—that is, providing light content designed to encourage men to communicate with others. The purpose of these topics was to deliver small items of relevant information to participants in an engaging manner and to encourage them to share information and support their peers by participating in the conversation. New content was added to the app twice a week, coinciding with a push notification being sent out to alert users.

### Study Aim

The aim of this qualitative study was to describe the way new and expecting fathers used the breastfeeding-focused conversation forum provided in the Milk Man app.

## Methods

### Sample

This study was part of a randomized controlled trial, the Parent Infant Feeding Initiative (PIFI; ACTRN12614000605695), which has been previously described [28]. The PIFI aimed to investigate the impact on breastfeeding duration of two different interventions, a male-facilitated antenatal class and the Milk Man app, both in isolation and combination. The project was approved by the Curtin University Human Research Ethics Committee (HR 82/2014; 14 May 2014).

Participants were recruited to the study through antenatal classes at maternity hospitals in Perth, Western Australia from August 2015 to December 2016. In total, 1426 couples were recruited to the study, with 681 couples being randomly assigned to an intervention arm that gave them access to Milk Man. Of these, 586 (86.0% [586/681]) went on to download the app. Reason for participants choosing not to participate in the PIFI study was not recorded.

### Procedures

After the study was explained, participants were issued a consent form, and upon consenting, were informed of the group they had been randomized into. As fathers signed up to the app on a rolling basis, conversation groups were started when there was a minimum of 5 participants with babies due in that month. Participants who commented at least once in the forum were included in this study. Data collected for this study include the period from antenatal signup to 26 weeks postpartum.

The Milk Man forum was moderated by the research team, and a set of management protocols was developed to govern the administration of the app. The protocols describe a hands-off approach to moderation, intervening only when certain criteria were reached. Among others, these criteria included if a contributor was using the forum to attack another user or if misinformation was being shared. In the event intervention was deemed necessary, a member of the research team who was a father of two young children assumed the role of *MacDaddy* to provide a peer response in consultation with another team member. The peer-dad responder was identifiable through his avatar (the Milk Man app logo) and username (MacDaddy), to ensure it was clear to participants that he was connected with the study, as opposed to another father participating in the trial.

### Data Analysis

All comments posted by participants were included in this data analysis. The data were then imported into NVivo 11 (QSR International) [29] and analyzed using a thematic analysis that involved coding the data into themes to enable organization and understanding of data [30]. Line-by-line analysis was used to examine words and phrases to explore the frequency, intensity, and extensiveness of discussion. Nodes were initially generated and then collapsed to form key themes. Data were coded manually and then checked by a second researcher trained in qualitative analysis to ensure conformability [31]. A comment could be allocated to multiple individual nodes depending on the content. For example, the following comment was coded to four individual nodes (Concern: not being good father, reflective parenting, sharing intimate information, and getting ready to be a dad):

One thing I fear about fatherhood is not being the best parent and role model. I’ve got pretty big shoes to fill as my parents were pretty amazing in their approach!

## Results

### Participant Demographics

A total of 586 participants signed up to the Milk Man app. Of those, 208 (35.5%, 208/586), hereafter known as contributors, posted at least once in the forum. Demographic information was available for 187 of these contributors (baseline questionnaire not completed by 21 individuals). Most contributors were in the age range of 30 to 34 years (47.1%, 88/187), had some university education (60.4%, 113/187), and approximately two-thirds were born in Australia (66.8%, 125/187). Table 1 describes the contributor characteristics. Contributors reflected Australian demographics in age (median age of men becoming fathers was 33.3 years in 2016) [32] and country of birth (67% of the population were born in Australia) [33]. However, they were more highly educated (28% of Australian men in the age range of 20-64 years have completed a Bachelor degree or higher) [34].

### Qualitative Analysis of the Conversation Forum

A total of 1493 comments were posted in the forum from the 208 contributors. In addition, there were four comments posted by MacDaddy in response to fathers sharing misinformation. The comments posted by the research team mainly provided correct information and links for Milk Man users. These 1497 comments were assigned to a total 54 tree nodes, generating a total of 3799 individual nodes (comments could be assigned to multiple nodes). These 54 tree nodes were then collapsed to form four key themes.

The number of comments posted per contributor ranged from one to 71. The average number of comments posted per contributor was 7.2 (mode 1; median 36). The number of comments per discussion topic ranged from one to 86 (average 24, mode 4, median 26). Participation was concentrated in the antenatal period and up to 6 weeks post birth, with approximately 80% of commenting activity happening within this time. Four main themes emerged from the data, and these and the subthemes describing the way fathers used the forum are presented in Table 2.

**Table 1:** Contributor characteristics (N=187).

Characteristic		n (%)
**Age in years**		
	<30	29 (15.5)
	30-34	88 (47.1)
	≥35	70 (37.4)
**Education**		
	High school or trade	72 (38.5)
	Some University education	113 (60.4)
**Country of birth**		
	Australia or New Zealand	125 (66.8)
	United Kingdom or Eire	22 (11.8)
	Africa or Middle East	12 (6.4)
	Asia	10 (5.3)
	Other	16 (8.6)

**Table 2:** Themes and subthemes of how fathers used the conversation forum in the Milk Man app.

Theme and subtheme		Example quote^a^
**Seek and offer support**		
	Support seeking	*My wife’s friend who has had a baby said to be flexible with a plan as breastfeeding often does not go to plan has anyone else heard people say this?*
	Support giving	[Responding to another user talking about the benefits of attending antenatal classes] *Yeh agreed! I gained allot more than anticipated tbh [to be honest]. Definitely recommend to up and coming future fathers :)*
	Supporting mums	*I’ve found just sitting with her while she’s breastfeeding is helping her. Doing small things like moving baby’s hand out of the way rubbing her back getting her water or a snack etc.*
	Health professional and other support	*I’ve learnt tonnes in all the antenatal classes looking forward to putting my knowledge to good use.*
**Social connection**		
	Joining in	[In reply to “What’s your best bloke outing?”] *Getting to footie.*
	Conversational	*I play soccer. so I’ll be keeping that up. great fitness and stress relief and catch up with friends after the match*.
	Using humor	[when discussing skin-to-skin with baby] *My wife suggested I trim the rug [chest hair] down!! I’ve spent a lifetime on this.*
Informational support		*Up until now my main contribution to reducing the housework load was a simple lowering of standards (only half kidding). We recently sat down and spelled out / wrote down some specifics to go on my plate like kitchen benches cleared and wiped every night so not waking up to a depressing site.*
**Sharing experiences**		
	Breastfeeding	*Just be supporting and encouraging will go a long way! I know If I give up my wife will give up on breastfeeding!*
	Fatherhood	*I’m looking forward to be a loving supportive encouraging Dad with an aim to assist in moulding a wonderful self-sufficient human being in the long run. AND I want to be a great friend to my child.*
	Sharing intimate information	*My son arrived last week and I can safely [say] words cannot describe how amazing it was and how proud I am of mum and Bub it really is an intense experience.*
	Bonding	*Had some skin-to-skin contact directly after my wife about 30 minutes after our son was born. An amazing feeling that I’ll cherish forever.*

^a^Quotes are reported verbatim as posted by the contributors.

### Seek and Offer Support

Fathers used the forum in several different ways relating to social support. This included using it to seek support, to offer support, to discuss how they were supporting their partners, as well as discussing other forms of support, including from professionals and other apps.

### Support-Seeking and Giving

Across a range of parenthood-related topics, fathers both sought and offered support within the forum. The giving of support, including offering tips and suggestions, was more common than fathers specifically seeking support. Support was offered directly in response to a request from another user, in response to a question posed within the app, or sometimes was unsolicited. The following comment is one example of a father offering unsolicited support to other fathers when discussing paternity leave (Fly In, Fly Out [FIFO] describes the shift work patterns of mining and oil or gas platform workers who work away from home):

[I am] Lucky with FIFO I will get 5 outa 6 weeks off so hopefully that works well before going back to the normal roster thinking the even time roster should work pretty well but you never know. Feel sorry for the boys who work the longer rosters away or fellas that can't have too much time off. Planning the flight home is the biggest gamble!!

Support-seeking was characterized by direct questions posed to the group, or users posting about a difficult experience, as illustrated in the following post:

It is day 3 since our bundle of joy arrived. my wife is struggling to get the milk flowing and the baby is not sucking hard enough. We were told that it takes up to 72 hours before milk flows which I didn’t know until the baby arrived. my worry is with bottle feed[ing] the baby seems to just easily get his feed. Will he choose to not work as hard when we try the breast and when should we say OK baby is hungry let’s feed him bottle? I don’t want my wife to feel as a failure if our desire to breastfeed fails. Any other fathers with similar dilemma?

There were instances of fathers seeking more support and connection from the app than they were able to receive. This includes fathers expressing a desire for real-life meetups to be organized, posting questions or comments and not getting a response in return, or expressing disappointment in the lack of conversation, as illustrated in the following post:

I tell you who makes woman depressed it’s the health nurses. She needs to put on weight or she has to be put on formula! She’s only a few grams lighter she was born 2 weeks early and she eats like her Daddy and [I] can never [gain] weight, they go by a stupid table. She’s nice and healthy. They tell you to eat healthy and then tell you [she’s] eating too many greens. These health nurses are useless! We doing pretty well. Shame we don’t have any family to help out here. No one wants to make friends on here? That’s the whole point of the App?

### Supporting Mums and Other Support

Contributors used the app to talk about how they were supporting their partners. This included discussions about breastfeeding, work, practical support, and mental health. One father in discussing the way he was getting ready to support his partner with breastfeeding posted the following:

Got a rocking chair with leg rest set up next to the window looking out over the streetscape facing a TV and a tower speaker connected to an old iPad with her favorite music. I think we’re ready!?

Fathers also posted examples of specific topics in the app prompting real-life conversations with their partners about how they could better support them, as illustrated in the following posts:

This is a good idea. I might talk to my wife about a breastfeeding plan. I would be keen to know how she wants my help.

I asked my wife what our plan was after reading this. Apparently if we get separated she would have already expressed milk and it won’t be an issue. Plan ahead and hope for the best is our plan I suppose!

### Social Connection

Topics posted to the app by the research team varied in their intent. The content areas, while with a focus on breastfeeding, were broad and included other parent-related issues such as sleep, relationship changes, starting solids, and bonding among others. Throughout the schedule of topics was an ongoing focus on community building. Ensuring there were topics that provided opportunity for light conversation and connection was deemed important in keeping fathers engaged and interested in the forum content.

A major emergent theme was that fathers used the forum as a way of connecting with other fathers and seeking companionship by participation. This was evident in the posts which did not relate to a particular health or parenting issue and simply reflected fathers *joining in* or creating conversation, often by using humor. Many fathers used humor when posting conversationally. This included recounting experiences, anticipating experiences, and merely joining in. In responding to a post asking what tips fathers may have for new dads, one contributor wrote the following:

Learn how to make her Vegemite on toast just right. It sounds like an easy job but f**k me I never knew you could get it wrong! Tip for rookies ensure the butter is melted in before the vegemite is applied 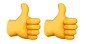
near death experience that one.

Another father shared this post when answering a topic asking if he was talking to his baby antenatally. He stated the following:

We would often fall asleep listening to an audio book. Our bub might think Stephen Fry is her father…

Posts coded as *joining in* included any time fathers came to the app to participate, rather than to specifically seek or share information. These were often shorter answers, yet these posts still reflect a commitment to participation and companionship. The following is an example of answers given to the “Who is the best celebrity father?” question:

The Pitt has got it wrapped up in the current landscape!

Has to be Phil Dunphy [Modern Family]. Everyone thinks I’ve modelled myself on him. Dad jokes just come natural for me though I’m still flattered.

Homer Simpson.

Hugh Hefner.

Surely George Foreman. Has 10 children (5 boys all named George and 5 girls). He found new and innovative ways to feed them and in the process created an empire of cooking appliances.

### Informational Support

Informational support has been defined as the provision of advice, suggestions, or information that will be useful to someone else [35]. The provision of this type of support through the app also created opportunities for observational learning by modeling a behavior or an attitude and was one of the four key ways fathers used the forum. Sometimes these posts were in response to specific questions and sometimes were fathers simply sharing what had worked for them. These differed from the other subthemes in that they were not directed toward seeking or offering support to another person and involved a father providing a personal statement. They all displayed opportunities for other fathers to learn from and for normalization of specific behaviors or attitudes. One example is from a topic asking whether fathers were planning on having skin-to-skin contact with their babies in hospital. The topic linked to an article in the library section of the app containing information about one father’s experience. The following posts provide an example of informational support:

I read an article on this app where the dad was the first skin to skin contact his baby received. Something to do with a complicated birth and having a caesarean. He went in prepared with a top he could un-button easily in fact. In the event of a tricky birth and if my Mrs wasn’t in a position to make that first contact for sure I’d love to be the first person my son meets!!!

Definitely keen to do skin to skin—or rather skin to chest rug—being more appropriate in my case.

I did skin to skin it was cool and helped relieve some stress of the birth when mum was taken to theatre.

This topic generated considerable discussion. The following examples are from fathers responding to the conversation and considering something they may not have thought of otherwise:

Hadn’t thought about dad/baby skin to skin. It makes sense that it could benefit the bonding experience.

Wow what a great read! Something for fathers right from baby’s first hours alive.

As research has demonstrated that some fathers report feeling uncomfortable about their partner breastfeeding in public, the app included content about this issue. The comments posted on this topic provide an example of how the forum provided opportunities for the normalization of public breastfeeding, which are as follows:

We have had no issues. Makes me think it really isn’t an issue.

My wife uses a shawl for a little discretion. She actually had a lady tell her that she shouldn’t have to cover up!

Pretty good, no issues or disagreeing public response.

### Sharing Experiences

Sharing experiences, both anticipatory and as reflections, emerged as a key way that fathers used the app forum. These experiences were broad, including a wide range of content areas, for example, breastfeeding, fatherhood, sleep, relationship changes, bonding, and mental health. One father stated the following:

We have just made it past week 2 but it has had some challenges especially the first week. Just need to persevere as it did get easier we got a lot of advice from the mid wives and you just need to figure out what is right for you and your new bub will pick it up. Just be supportive as the wife can get emotional during this.

Some of the information and experiences shared was of an intimate nature. The following post is from one father who is discussing how they announced their pregnancy:

We lost [our first] one. The emotional struggles after that meant telling people the second time wasn’t the same. All good now though. 34 weeks and the little one is fit as a malee bull!

Other posts were confessional and honest, as illustrated in the following quote:

I must admit I like being at work a lot more than being left [with] the baby by myself for an extended period. Talk about stressful!

## Discussion

### Principal Findings

The qualitative data reported in this paper demonstrate that fathers are prepared to use a breastfeeding-focused app-based forum. Contributors in this study used the forum in a variety of ways: to seek and offer support, to share experiences, to build connection, and to offer informational support. Some fathers used the app to share very personal information, including about miscarriage, resuming intimacy with their partner, and how fatherhood was making them feel. Others used it in a less intimate way, using it simply to join in, or to participate. This is an important finding, as even by contributors providing short comments, the commitment to seek companionship and to connect is evident by the completed action of writing and posting a comment.

An earlier study of an OHC found that discussion of topics other than the health issue were a key factor in retaining engagement [14]. The subtheme of users seeking connection by joining in suggest this may be a factor in this study as well. This is an interesting finding as although off-topic discussions may be viewed as irrelevant, including and encouraging this type of conversation and posting in an OHC may be a key component of sustained engagement. The relative anonymity of the online forum may have made fathers feel more comfortable participating in the conversation.

The Milk Man forum was a researcher-facilitated forum, in that fathers were encouraged to respond to questions posed by the research team. Naturally, this has guided the content covered by the posts. Approximately a third of users with access to the Milk Man app commented in the forum. This is a higher percentage than has been observed in other studies and is further validation of this approach with new and expecting fathers. For instance, in exploring interaction with OHCs, some researchers have described a 90-9-1 principle [36,37]. This principle observes that 90% of users are lurkers who observe but never post, 9% contribute a small amount of content, and 1% of users contribute most of the activity in the forum [37]. To investigate if this rule applied to digital health behavior change interventions, a study was carried out with four OHCs (based on alcohol, depression, panic, and smoking cessation) [36]. Across the four OHCs, there were 578,349 posts and 63,990 users. The authors found that overall, less than 25% of users posted at least once in a forum. Usage patterns were consistent with the 90-9-1 rule, with an average of 73.6% (59%-75%) of the content being generated by the top 1% of users, an average of 24.7% (17.3%-24.7%) by the next 9%, and the remaining 90% contributing an average of just 1.7% (1.1%-7.8%) [36]. Similar to these findings, a breastfeeding app for mothers with a sharing function found that 14% of their app users’ commented at least once [38]. Other participants used the Milk Man app in different ways, and further evaluation is in progress of the benefit these noncontributors, or lurkers, received from the forum.

Most activity in the forum occurred between when fathers first signed up to the app (antenatally) and 6 weeks post the birth of their child. Content continued to be added to the conversation up to 26 weeks post birth. It is unclear if the drop-off in posting activity was because of reduced activity in the forum, or if that is the natural time that fathers would use the app for. There were examples of fathers wanting more support and connection from the app than they received. Due to the relatively small size of the conversation groups (average 32 participants, range: 16-47), participants may have been dissuaded to continue posting in the forum as momentum declined over time. More meaningful interaction between fathers, including more genuine conversation, may be achieved with a bigger cohort. It is important to note that participants in different groups will have had different experiences with participation in the forum as some groups were significantly more active than others, and users could only view the content in their own group. Further evaluation of the app and the wider project, including breastfeeding and other outcomes, is currently underway and will provide further insight into app engagement.

The research team took a very hands-off approach to the forum, intervening only when the management protocols required it. As there were examples of fathers wanting more support from the app than they received and that activity dropped off after 6 weeks postpartum, it would be valuable to examine the impact of an increased level of researcher interaction in the forum. This could include through the implementation of a peer-based coaching program embedded within the app. Peer mentors could help get discussions going, could lead conversations with fathers, and provide individualized support, and future research can investigate the impact this has on the way fathers use the app and their engagement with it. Other studies have found that participants can highly value professional moderation and feel that it can help create vibrant communities, provide information, and help with solutions [39].

The way this study has found how fathers used the forum will be useful in informing the development of strategies designed to engage participants in digital social support interventions. Researchers can create content designed to enhance opportunities for fathers to communicate in the way this study has described. For example, facilitating specific opportunities for fathers to broadly discuss different mechanisms of support and creating spaces for the sharing of intimate information. Additionally, including light, conversational-driven content may increase forum activity and the number of app users contributing in the conversation forum. Further research with a larger sample size and alterations to the forum to increase connectively will be of value in further determining the impact online forums can have on engaging fathers with breastfeeding information and support.

### Strengths and Limitations

This study had some clear limitations. The forum discussion was researcher led and limited to topics posted by the research team. A decision was made to limit the conversation to content posed by the research team, and fathers were not able to post their own content for other fathers to comment on. The research team considered this function in the planning phase, however, determined there was a potential risk in topics being poorly informed or containing inaccurate or misleading information. Allowing this function may change the way fathers use the forum. Due to the way participants were grouped, the number of fathers in a conversation group were relatively small. Participants from across the study will have had different experiences of the forum as usage differed between groups, and users could only see content in their own group. This study consisted of a qualitative study looking at how fathers used the app. Further investigation, including consideration of the 90-9-1 principle and identifying key influencers, will be a focus of future research. Data from the app analytics framework are also currently being prepared for publication. The strength of this study is that this is the first paper we are aware of to report on the way fathers use a conversation forum about breastfeeding. Although there were limitations, the interaction reported in this study points to this being an area that requires further exploration as a way of supporting fathers.

### Conclusions

Research has shown that fathers value peer support in the perinatal period, and this research adds to that evidence, including, importantly, that fathers are prepared to access that support online through a mobile app. Fathers have an important role to play in supporting their partners with breastfeeding; however, they are rarely a key target group for antenatal education and support services and are often a hard group to reach. To better support fathers in this important time in their life, as well as increase their support for their partners, it is that vital innovative ways to reach parents are explored. This paper demonstrates that an app-based online forum delivering parenting and breastfeeding information is an acceptable method and one in which fathers were prepared to use to share information and display supportive behaviors.

There remains more that can be done in terms of research with this hard to reach group. Future directions include conducting research on a population level with a larger sample, including more interactive features and investigating the impact peer coaching has on utilization of the app. This paper adds to the evidence on how to reach fathers in the perinatal period and discusses the different ways fathers use an app-based forum. This research will be of interest to anyone seeking to reach fathers in this critical period.

## Abbreviations

OHC: online health community
PIFI: Parent Infant Feeding Initiative
